# A Standard Protocol for the Calibration of Capillary Electrophoresis (CE) Equipment

**DOI:** 10.3797/scipharm.1109-23

**Published:** 2011-10-06

**Authors:** Bodo Lachmann, Hannelore Kopelent-Frank, Christian R. Noe

**Affiliations:** Department of Medicinal Chemistry, University of Vienna, Althanstraße 14, 1090 Vienna, Austria

**Keywords:** Capillary electrophoresis, Calibration of analytical instruments, GLP, ICH

## Abstract

Calibration of complex analytical systems is always a difficult task. Nevertheless, a suitable approach has to be designed before the systems can be introduced into routine analysis. In literature, many methods have been described for the purpose of calibrating such systems, but only a few of them deal with capillary elctrophoresis. Here, we want to demonstrate a general approach to how the calibration of this type of analytical instrument becomes feasible.

## Introduction

In accordance to the EU-GMP guide [1] and several ICH guidelines [2], dealing with the topic of pharmaceutical analysis, calibration of analytical equipment is one of the “must” topics prior to the validation of analytical methods. In most cases, this takes place after successfully performed Design- (DQ), Installation- (IQ) and Operation Qualification (OQ). During these phases, it is demonstrated that the equipment meets the user requirements, that the equipment is appropriately installed and it is proven, and that the equipment operates within its predetermined ranges. After these basic qualification activities, a performance qualification (PQ) should take place, which shows that the equipment is able to fulfil its intended use. Part of these PQ procedures could be a first calibration of the instrument leading to a combined calibration/performance qualification report. In addition, calibration is also part of the requalification activities, performed after a defined time period in accordance to the rules of good laboratory practice.

In this contribution we want to introduce a generalised approach to how CE equipment could be calibrated. Based on a standard operating procedure (SOP) a calibration has been performed, controlling several instrumental parameters such as temperature, current stability, reproducibility of the injection system and standard deviation of peak areas and migration time, with and without internal standard. Contrary to the performance tests of most suppliers, we have used two different buffer systems to check the above mentioned parameters, e. g. buffer A at pH of 9.3 (sodium tetraborate, high EOF) and buffer B at low pH (triethyl amine pH 2.0 adjusted with phosphoric acid), suppressing the EOF nearly completely, leading to results, which are more comparable to the “normal” operation conditions than the supplier tests. In addition, peak areas and migration times are strongly influenced on the “fitness” of the capillary used, due to the fact that the migration times in an alkaline buffer system are extremely dependent on EOF, which is not the case at low pH values.

**Fig. 1:**
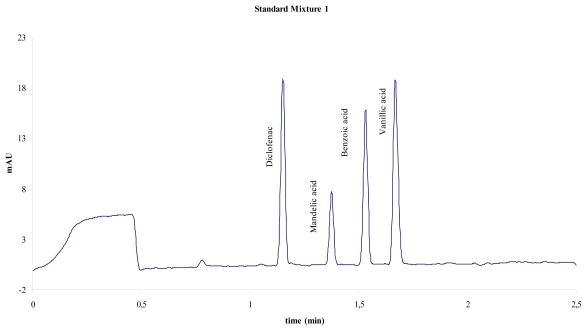
Electropherogram of mixture 1, 25 mM sodium tetraborate, pH 9.3; 25 kV, 25 °C

**Fig. 2:**
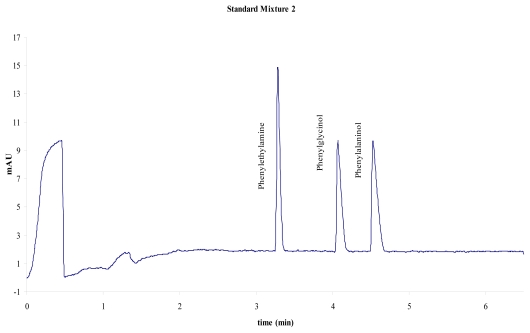
Electropherogram of mixture 2, 66 mM triethyl amine, pH 2,0; 25 kV, 25 °C

For calibration purposes, two mixtures of standard substances have been used: one mixture of four aromatic acids (system 1, fig. 1) and a mixture of three aromatic amines (system 2, fig. 2). The following table (tab. 1) summarises the activities suggested for the instrument calibration:

## Results and Discussion

**Fig. 3:**
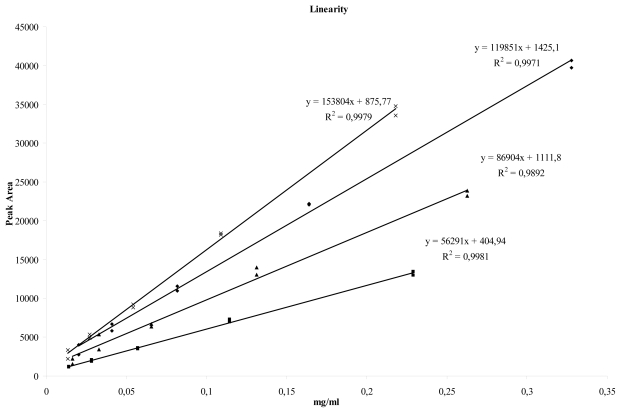
Linearity data of system 1, ◆: Diclofenac, x: Vanillic acid, ▴: Benzoic acid, ■ Mandelic acid

The following two figures show typical electropherograms obtained with both test mixtures used:

Although validation kits for CE methods are available [3] from commercial sources and generalized descriptions for the validation of CE methods have been published years ago [4, 5], we decided to develop and to perform an in-house calibration program for our CE-equipment. As generally accepted, reproducibility and sensitivity in capillary electrophoresis is not as good as in HPLC. The criteria of acceptance of our program in comparison to standard calibration procedures for HPLC-equipment have been expanded. However, it has been shown that reproducibility can be poor without internal standard (see table 2, results for system 2). Keeping this in mind, the obtained correlation coefficients of the linearity testing should be evaluated critically as well (see Fig. 3).

As mentioned in the literature over 10 years ago [6], the injection system is the main source of error in quantitative CE, not only due to the small amounts injected, but also because of the type of the injection system. Nevertheless, using an internal standard, comparable quantitative results as in HPLC can be obtained, as shown by our data, in combination with a separation efficiency which can not be exceeded by any other analytical separation technique and with an extremely fast method development process. Further work will be focusing on the optimization of our protocol and on the application of this protocol to CE-units from different manufactures.

## Experimental

### Equipment

All separations were performed using a P/ACE MDQ capillary electrophoresis system with an UV detector (fixed wavelength) (Beckman Instruments, Munich, Germany). Photometric on-column detection was carried out at 214 nm. 32 Karat^©^ software was applied for instrument control, data acquisition and analysis.

### Electrophoretic conditions

Uncoated fused-silica capillaries (Polymicron) of 50 μm I. D. (385 μm O. D.) with polyimide coating of the outer surface were used for all separations. Capillaries of a total length of 30 cm were used and the detector was situated 10 cm (PACE MDQ) from the cathodic end (normal polarity). The capillary was flushed with 0.1 M NaOH for 1 min and with buffer for further 1 min prior to each analysis, in addition with 0.1 M NaOH for 20 min prior to first use. Samples were injected by pressure (0.5 psi) and separations were carried out with 25 kV at ambient temperature (25 °C) (Beckman capillary cartridge coolant).

### Chemicals and Buffers

All chemicals were purchased from Sigma-Aldrich and Acros. Separation buffer 1 was prepared by diluting a 0.1 M stock solution of sodium tetraborate, resulting in a concentration of 25 mM sodium tetratborate, pH 9,3. Separation buffer 2 was prepared by solving 1 g of triethyl amine in 150 ml of HPLC grade water and adjusting pH 2.0 by adding 85 % phosphoric acid.

### Samples

The two test mixtures were prepared from stock solution of the following concentrations:

#### Mixture 1

Diclofenac Sodium (DF) 131,1 mg/100 ml, Benzoic acid (BA) 105,1 mg/100 ml, Mandelic acid (MA) 22,9 mg/25 ml and Vanillic acid (VA) 21,8 mg/25 ml. The substances have been solved in a mixture of BGE and water (2+8). 100 μl of each solution has been used for the standard mixture.

#### Mixture 2

Phenylethylamine (PEA) 25,2 mg/25 ml, Phenylglycinol (PGL) 19,5 mg/25 ml and Phenylalaninol (PAL) 20,1 mg/25 ml. The substances have been solved in water with addition of 5 ml of 0.1 N HCl. 100 μl of each solution has been used for the standard mixture.

## Figures and Tables

**Tab. 1:** Description of planned calibration activities

Calibration Parameter	Acceptance criteria	Test system
Reproducibility of:		
Peak Area / Migrationtime (without I.S.)	RSD less than 5% / 5 %	System 1 and System 2, 6 replicates
Peak Area / Migrationtime (with I.S.)	RSD less than 3% / 3 %	
Sample contamination / cross contamination / carry over	Peak area less than 1 % of the peak area of the standard solution	System 1, injection of a blank after injection of the standard solution
Linearity of the injection system	R not less than 0.99	Mandelic acid, injection times from 3 to 10 secondes
Linearity of the current	R not less than 0.99	System 1, voltage applied from 15 to 30 kV
Current stability over 3 min	Not more than 2 % difference	System 2, at 25 kV
Sensitivity of the detection system	S/N not less than 25	Series of dilutions, system 1
Temperature stability	No change over 1 °C	System 1, system 2
Linearity of the peak areas	R not less than 0,99	System 1

Calibration Parameters (I.S.: one of the components used as internal standard, RSD: relative standard deviation, S/N: Signal to Noise Ratio)

**Tab. 2:** Calibration results:

Calibration Parameter	Results	Comments
Reproducibility of:		
System 1		
Peak Area (without I.S.)	RSD: DF 4,03 %, MA 4,76 %, BA 4,02 %, VA 3,71 %	Passed (1)
Migrationtime (without I.S.)	RSD: DF 0,21 %, MA 0,27 %, BA 0,36 %, VA 1,32 %	Passed
Peak Area (with I.S.)	RSD: DF 1,57 %, MA 1,29 %, BA n.a. (2), VA 1,42 %	Passed (1)
Migrationtime (with I.S.)	RSD: DF 0,19 %, MA 0,13 %, BA n.a. (2), VA 1,65 %	Passed
System 2		
Peak Area (without I.S.)	RSD: PEA 13,1 %, PGL 13,4 %, PAL 13,2 %	Failed (3)
Migrationtime (without I.S.)	RSD: PEA 1,04 %, PGL 1,56 %, PAL 1,13 %	Passed
Peak Area (with I.S.)	RSD: PEA 0,76 %, PGL n.a. (2), PAL 0,63 %	Passed
Migrationtime (with I.S.)	RSD: PEA 0,64 %, PGL n.a. (2), PAL 0,54 %	Passed
Sample contamination / cross contamination / carry over	None of the four substances was detectable in the blank	Passed
Linearity of the injection system	R = 0,9904 for Mandelic acid	Passed
Linearity of the current	R = 0,9977 between 15 and 30 kV	Passed
Current stability over 3 min	Not more than 0.62 %	Passed
Sensitivity of the detection system	S/N higher than 30 for dilution 5	Passed
Temperature stability	stable	Passed
	see Figure 3 (4)	
	DF: R = 0,9985	Passed
Linearity of the peak areas	MA: R = 0,9990	Passed
	BA: R = 0,9946	Passed
	VA: R = 0,9985	Passed

Table 2: Calibration results, (1) after elimination of an outlier MA, (2) not applicable, used as internal standard, (3) last (6th) injection results in 20 % higher peak areas, compared with the first five injections, (4) calibration curves see fig. 3.
